# In Silico Design of a New Epitope-Based Vaccine against Grass Group 1 Allergens

**DOI:** 10.3390/arm91060036

**Published:** 2023-11-08

**Authors:** Dzhemal Moten, Tsvetelina Batsalova, Desislava Apostolova, Tsvetelina Mladenova, Balik Dzhambazov, Ivanka Teneva

**Affiliations:** 1 Department of Developmental Biology, Faculty of Biology, Paisii Hilendarski University of Plovdiv, 24 Tsar Assen Str., 4000 Plovdiv, Bulgaria; moten@uni-plovdiv.bg (D.M.); tsvetelina@uni-plovdiv.bg (T.B.); apostolova@uni-plovdiv.bg (D.A.); balik@uni-plovdiv.bg (B.D.); 2 Department of Botany and Biological Education, Faculty of Biology, Paisii Hilendarski University of Plovdiv, 24 Tsar Assen Str., 4000 Plovdiv, Bulgaria; cmladenova@uni-plovdiv.bg

**Keywords:** pollen allergy, grass allergens, immunoinformatics, in silico, epitopes, peptide-based vaccine, ex vivo, immunotherapy

## Abstract

**Highlights:**

**What are the main findings?**

- A peptide consisting of 20 amino acids was designed in silico as a grass pollen allergy vaccine.
- The epitope-based vaccine has been validated by molecular docking analysis and ex vivo T-cell stimulation assay. It covers more than 80% of the European and global population.

**What is the implication of the main finding?**

- The immunogenic peptide can be used for treatment of patients with grass pollen allergy by triggering the T-cell response and production of competitive IgG antibodies.
- Additional studies are needed before the clinical application of the epitope-based vaccine for immunotherapy.

**Abstract:**

Allergic diseases are a global public health problem that affects up to 30% of the population in industrialized societies. More than 40% of allergic patients suffer from grass pollen allergy. Grass pollen allergens of group 1 and group 5 are the major allergens, since they induce allergic reactions in patients at high rates. In this study, we used immunoinformatic approaches to design an effective epitope-based vaccine against the grass group 1 allergens. After the alignment of all known pollen T-cell and B-cell epitopes from pollen allergens available in the public databases, the epitope GTKSEVEDVIPEGWKADTSY was identified as the most suitable for further analyses. The target sequence was subjected to immunoinformatics analyses to predict antigenic T-cell and B-cell epitopes. Population coverage analysis was performed for CD8+ and CD4+ T-cell epitopes. The selected T-cell epitopes (VEDVIPEGW and TKSEVEDVIPEGWKA) covered 78.87% and 98.20% of the global population and 84.57% and 99.86% of the population of Europe. Selected CD8+, CD4+ T-cell and B-cell epitopes have been validated by molecular docking analysis. CD8+ and CD4+ T-cell epitopes showed a very strong binding affinity to major histocompatibility complex (MHC) class I (MHC I) molecules and MHC class II (MHC II) molecules with global energy scores of −72.1 kcal/mol and −89.59 kcal/mol, respectively. The human IgE-Fc (PDB ID 4J4P) showed a lower affinity with B-cell epitope (ΔG = −34.4 kcal/mol), while the Phl p 2-specific human IgE Fab (PDB ID 2VXQ) had the lowest binding with the B-cell epitope (ΔG = −29.9 kcal/mol). Our immunoinformatics results demonstrated that the peptide GTKSEVEDVIPEGWKADTSY could stimulate the immune system and we performed ex vivo tests showed that the investigated epitope activates T cells isolated from patients with grass pollen allergy, but it is not recognized by IgE antibodies specific for grass pollen allergens. This confirms the importance of such studies to establish universal epitopes to serve as a basis for developing an effective vaccine against a particular group of allergens. Further in vivo studies are needed to validate the effectiveness of such a vaccine against grass pollen allergens.

## 1. Introduction

Allergic diseases are chronic, inflammatory disorders with aberrant immune reactions to theoretically innocuous environmental antigens, which are called allergens [1]. Allergy is a global health problem that affects up to 30% of the population in industrialized countries and it can be compared with other major diseases affecting humankind such as cardiovascular diseases, autoimmune diseases, cancer, and metabolic diseases. According to data of the European Academy of Allergy and Clinical Immunology (EAACI), about 21–32% of the population suffer from pollen allergens [2,3]. The constant increase in the number of pollen-sensitive patients observed in recent years depends on atopic genetic origin, as well as natural allergen exposure and other environmental factors [4]. So far, 1095 different allergens have been officially described, of which 240 are registered as plant-derived airborne allergens (https://www.allergen.org, accessed on 1 March 2023).

Allergenic pollen comes from three main plant groups—trees, grasses, and weeds. Allergens from these plant groups belong to diverse protein classes, the most significant of which are Bet v 1 homologs, β-expansins, profilins, lipid transfer proteins (LTPs), polcalcins, and group 5 allergens [5]. Allergens from grass pollens are the most frequent elicitors of IgE-mediated allergies in many parts of the world, provoking allergic symptoms such as rhinitis, allergic asthma, conjunctivitis, and atopic dermatitis [6,7].

Most allergenic grasses belong to the family Poaceae (Gramineae). The Poaceae family is composed of more than 600 genera and 12,000 species and its members are widespread nearly everywhere throughout the world [8]. A limited number of grass species are recognized as major causes of allergic diseases. At present, 13 different groups of allergens related to grass pollen were identified and characterized from one or more species. These groups represent a variety of glycosylated and non-glycosylated proteins with different structures, sizes, and physicochemical properties [9]. Out of these, group 1 and group 5 allergens were found to be the most clinically relevant, since they induce allergic reactions in patients at high rates (65–85% of patients allergic to grass pollen are sensitive to group 5 and 90–95% to group 1 allergens), while other allergens, such as groups 2, 3, and 4, were recognized by 80, 60–70, and 75%, respectively [10,11,12]. All group 1 allergens have been categorized as a subclass of the beta-expansin family (60–70% sequence identity), a family of proteins involved in cell wall loosening and extension in plants through their activity of unlocking wall microfibril and matrix polysaccharide structures [13]. The group 1 allergens are glycoproteins with a molecular weight of 31–35 kDa and have two domains. The C-terminal domain appears to be immunodominant in terms of induction of allergen-specific IgE in humans. They contain seven conserved cysteine residues, located mainly in the N-terminal half of the polypeptide [14].

In order to develop an allergic reaction to a certain allergenic pollen protein, it is necessary that this protein to bind to the major histocompatibility complex (MHC) molecules expressed on the surface of the professional antigen-presenting cells (dendritic cells, macrophages, B cells), to be processed intracellularly and presented to the T cells such as an MHC–peptide complex [15]. In humans, MHC molecules are coded from human leukocyte antigen (HLA) genes, which are highly polymorphic. This presentation of the allergenic epitope by MHC proteins causing priming of the T cells and subsequent production of allergen-specific immunoglobulins (IgE) from the B cells is known as the first (sensitization) phase of the allergic reaction [16]. The second phase (elicitation) starts upon a second encounter of the allergen, inducing a chronic inflammatory reaction [16]. This interaction activates mast cells and basophils through binding of the allergen to the complex of the receptor for FcϵRI and IgE (FcϵRI-IgE) formed on the surface of these cells, resulting in degranulation and release of histamine, leukotrienes, prostaglandins, serotonin, and tryptase [16].

MHC class I molecules present to T cells short peptides (8–11 amino acids), while MHC class II may present longer peptides (12–25 amino acids) [17]. Normally, T-cell epitopes (recognized by T-cell receptors) and B-cell epitopes (recognized by IgE antibodies) on the pollen allergenic proteins are different. IgE antibodies preferentially recognize three-dimensional conformation of the allergenic protein molecule, whereas T cells recognize short linear epitopes presented by the MHC [15,18]. Therefore, short peptides that contain dominant T-cell epitopes of major allergens can be used to safely modulate allergen-specific T-cell responses inducing immunological tolerance.

The computational vaccine design work on genomic data uses a set of computational methods, including mathematical, chemical, and biological approaches to identify the most suitable epitopes for experimental testing. An appropriate selection of epitopes and corresponding peptides for therapeutic vaccines is crucial for success. For example, polymorphism of the genes encoding human MHC class I and class II presents a challenge for peptide vaccine design [19,20,21].

The progress in bioinformatics and immunoinformatics has been rapid and has led to the development of a variety of tools for the design of epitope-based vaccines in the postgenomic era [22,23]. In general, the steps involved in vaccine design using in silico methods include searching antigen protein databases, characterizing the epitopes recognized by both T cells and B cells, analyzing protein interactions (molecular docking and molecular dynamic simulation), and analyzing antigenicity and homology [23]. The goal of allergy vaccines is to induce a therapeutic immune response against the corresponding allergens. Overall, peptide therapy has been shown to reduce allergen sensitization and downregulate allergen-specific proliferative and cytokine responses in the blood [24].

Current pharmacologic treatments for allergic disorders are largely palliative (rather than curative). The only option offering a disease-modifying intervention is “allergen-specific immunotherapy” or an “allergy vaccine”, that directly interferes with the immune reactions causing the disease [25,26]. This study aims to design an efficient epitope-based peptide vaccine against the grass group 1 allergens (Lol-p1, Fes-p1, Phl-p1, Poa-p1, Sec-c1, Zea-m1, Ant-o1, Cyn-d1, Dac-g1, Hol-l1) by using various immunoinformatic platforms. Such a vaccine, composed of a short peptide that includes T-cell epitopes of several allergens, makes it possible to induce immunological tolerance in many patients (with different HLA haplotypes) allergic to a certain group of allergens without activating the typical allergic reaction, since there is no interaction with IgE antibodies. In this regard, epitope-based vaccines offer an outstanding opportunity and potential for the treatment of allergy.

## 2. Materials and Methods

### 2.1. Pollen T-Cell and B-Cell Epitope Sequence Retrieval, Multiple Sequence Alignment, and Phylogenetic Analysis

Several bioinformatics methods were used in this study for developing an epitope-based vaccine against the grass group 1 allergens. All known pollen T-cell and B-cell epitopes from completely or partially sequenced pollen allergens available in the public databases (Immune Epitope Database, Allergome, Allergen Database for Food Safety, UniProt, Allergen Nomenclature, Allergen Online, Structural Database of Allergenic Proteins, National Center for Biotechnology Information, accessed on 10 August 2023) were retrieved in fasta format.

To identify the conserved regions of pollen epitopes, a multiple sequence alignment was performed via ClustalW using molecular evolutionary genetics analysis software Mega X [27,28]. Selected sequences were aligned with other amino acid sequences of allergenic as well as non-allergenic proteins using the BLAST program of the NCBI (http://blast.ncbi.nlm.nih.gov, accessed on 16 August 2023) with parameters optimized for short query sequences (E-value threshold 100, low-complexity filter off, ungapped alignment for fragments shorter than 20 residues). A phylogenetic analysis was performed to visualize relationships between the allergenic peptides and other proteins. This analysis was conducted because the etiology of many allergic diseases is explained by previous bacterial infection and molecular mimicry of bacterial proteins to some allergens (including pollen allergens), which leads to cross-reactivity and development of allergic reactions. The phylogenetic tree was constructed through the maximum likelihood (ML) method (GTR + I + G evolutionary model) with 1000 bootstrap replicates by using Mega X [28].

### 2.2. Prediction of CD8+ T-Cell Epitopes and Their MHC-I-Binding Alleles

The MHC I prediction online server (http://tools.iedb.org/mhci, accessed on 18 August 2023) [29] of the Immune Epitope Database and Analysis Resource (IEDB) was utilized to predict MHC-I-binding epitopes of the target sequence. CD8+ T-cell epitopes were recognized by consensus approaches using various methods with default settings including an artificial neural network (ANN) [30] and stabilized matrix method (SMM) [31]. The selected sequence was submitted in fasta format. A complete human leukocyte antigen (HLA) reference set was used. The resulting epitopes were sorted according to predicted percentile rank, and five epitopes that showed the lowest percentile rank were selected.

### 2.3. Prediction of CD4+ T-Cell Epitopes and Their MHC-II-Binding Alleles

Identification of the CD4+ T-cell epitopes is one of the critical steps in developing an effective epitope-based vaccine. In the case of predicting helper T-cell epitopes of the target sequence, we used the MHC II prediction tool of the immune epitope database (IEDB; http://tools.iedb.org/mhcii/, with 85% accuracy, accessed on 3 October 2023). MHC-II-binding prediction was achieved using default settings of the NetMHCIIpan 4.1 EL method [32,33,34,35]. It covers the three human HLA-DR, HLA-DQ, and HLA-DP alleles. The epitope length was specified to be 15 mer. The epitopes with the lowest percentile rank represent a high affinity of MHC II.

### 2.4. Population Coverage Prediction for CD8+ and CD4+ T-Cell Epitopes and Their Alleles

Due to the polymorphism, MHC molecules can show great diversity among people of different countries or ethnicities. The population coverage analysis of all finally predicted CD8+ and CD4+ T-cell epitopes and their MHC I and MHC II molecules was assessed for the European and world population using the IEDB population coverage web server (http://tools.iedb.org/population/, accessed on 3 October 2023) [36].

### 2.5. Identification of B-Cell Epitopes

Potential linear B-cell epitopes that can induce the B cells to elicit antibody production were found using B-cell epitope prediction tools (http://tools.iedb.org/bcell/, accessed on 19 August 2023) of the Immune Epitope Database. This online web server uses different prediction methods such as Parker hydrophilicity prediction [37], Bepipred linear epitope prediction [38], Emini surface accessibility prediction [39], Kolaskar and Tongaonkar antigenicity scale [40], Karplus and Schulz flexibility prediction [41], and Chou and Fashman beta turn prediction tool [42]. The epitope with the best score was chosen for molecular docking analysis.

### 2.6. Molecular Interaction Analysis

Molecular docking is one of the most important approaches to design an effective peptide-based vaccine. Docking of the epitopes to their respective alleles was performed to determine whether our predicted epitopes can be presented on the cell surface by MHC molecules to elicit the recognition by appropriate T cells. Firstly, the crystallographic structure of HLA-B*44:03 (PDB ID 4JQX) and HLA-DQA1*03:01/DQB1*02:01 (PDB ID 4D8P) was retrieved from the RCSB protein data bank (http://www.rcsb.org/pdb/, accessed on 26 August 2023) to act as receptors for MHC I and MHC II epitopes, respectively. The 3D structures of the human IgE-Fc (PDB ID 4J4P) and Phl p 2-specific human IgE Fab (PDB ID 2VXQ) were docked with the predicted B-cell epitope. The immune receptors were prepared before running protein–peptide docking by removing associated ligands, water molecules, and heteroatoms. All of these procedures were carried out using the Discovery Studio Visualizer program [43]. In the next step, UCSF Chimera 1.14 was used for energy minimization for the structures [44]. The docking study was performed through the CABS-dock web server applying default settings [45]. We provide both the crystal structures of MHC molecules (MHC I and MHC II) and immunoglobulin E (IgE) in PDB format, while the ligands are in their fasta format. Each independent CABS-dock simulation run generated 10 top-scored models (10 top-scored models were selected on the basis of the maximum cluster size and the lowest energy score). Results of molecular docking were further refined via FireDock (http://bioinfo3d.cs.tau.ac.il/FireDock/, accessed on 26 August 2023) [46]. Complexes with the lowest global energy (most negative) were nominated for analyzing their molecular interaction patterns. The interactions of the candidate ligands with their immune receptors were analyzed and visualized by Discovery Studio Visualizer [43] and PyMOL [47], respectively.

### 2.7. Participants, Functional Validation of T-Cell Reactivity, and Antibody Recognition

Eight patients with proven allergy to grass pollen and ten healthy controls were recruited for this study. All participants were females and age-matched (36–44 years old). Allergic patients were selected in collaboration with an experienced allergist based on their case history: established allergy to grass pollen at least two years ago, a positive prick test to grass pollen (SOLUPRICK SQ, ALK-Abelló A/S Hørsholm, Denmark), and detected IgE antibodies specific to grass pollen (ImmunoCAP™ Grass Pollen Allergens, Thermo Fisher Scientific, Phadia AB, Uppsala, Sweden). Recruited healthy subjects had negative skin prick tests against pollen allergens isolated from the grass species *Lolium perenne*, *Phleum pratense*, *Holcus lanatus*, *Dactylis glomerata*, *Festuca elatior*, and *Poa pratensis*, against birch pollen (*Betula pendula*), mugwort pollen (*Artemisia vulgaris*), and house dust mite (*Dermatophagoides pteronyssinus*), and no clinical histories of pollen allergies. None of the recruited participants had received corticosteroids, allergen-specific immunotherapy, or antihistamines during the last 3 months before the sampling. All participants provided written informed consent according to the Declaration of Helsinki ethical guidelines under protocols approved by the Local Ethical Committee at the Paisii Hilendarski University of Plovdiv under No. 5/10.06.2020.

Peripheral blood samples were collected into BD Vacutainer^®^ K2EDTA tubes (Becton, Dickinson and Company, Oakville, ON, Canada) by a certified nurse and centrifuged at 1500× *g* for 15 min at room temperature. The sera were transferred into 2 mL microtubes and used for measurement of the antibody levels by ELISA. Red blood cells were lysed with 0.84% NH_4_Cl buffer, and after washing with a sterile Dulbecco’s phosphate-buffered saline (D-PBS, Gibco^®^, Life Technologies™, Paisley, Scotland, UK), the remaining cells were resuspended in RPMI 1640 medium, supplemented with 10% fetal bovine serum (FBS) and antibiotic–antimycotic solution (complete medium) (all from Merck KGaA, Darmstadt, Germany).

Cells were plated at a concentration of 1 × 10^6^ cells/mL in 96-well plates (TPP, Trasadingen, Switzerland), stimulated in triplicate with 10 µg/mL synthetic peptide (GTKSEVEDVIPEGWKADTSY, Schafer-N, Copenhagen, Denmark) and 1 μg/mL phytohemoagglutinin (PHA-L, Merck KGaA, Darmstadt, Germany) as a positive control or medium alone (negative control), and cultured for 5 days at 37 °C and 5% CO_2_ in a volume of 200 μL complete medium. After 5 days of stimulation, the production of IL-2 in the supernatants was measured by a LEGEND MAX™ Human IL-2 kit (BioLegend^®^, San Diego, CA, USA) according to the manufacturer’s instructions. The absorbance was measured at 450 nm using a SpectraMax i3x Multi-Mode Microplate Reader (Molecular Devices LLC, San Jose, CA, USA).

To assess whether grass-pollen-specific IgE antibodies within the sera of the allergic patients recognize the epitope-designed synthetic peptide GTKSEVEDVIPEGWKADTSY, we have performed ELISA tests. A grass pollen extract group (BB-NCIPD Ltd., Sofia, Bulgaria) containing 14 species (*Dactylis glomerata*, *Festuca* sp., *Lolium perenne*, *Secale cereal*, *Phleum pretense*, *Poa* sp., *Holcus lanatus*, *Agrostis alba*, *Bromus* sp., *Alopecurus* sp., *Agropyron repens*, *Arrhenatherum elatius*, *Zea mays*, and *Deschampsia caespitosa*) was used as a positive control. Corning^®^ 96 Well ELISA plates (Merck KGaA, Darmstadt, Germany) were coated overnight at 4 °C with 10 μg/mL of the synthetic peptide (50 μL/well). Unspecific binding was blocked with 5% heat-inactivated FBS in D-PBS for one hour at room temperature (RT) and plates were washed three times with ELISA buffer (D-PBS supplemented with 0.05% Tween 20). The serum samples from allergic patients and healthy individuals (diluted 1:5 in ELISA buffer) were added to the coated plates (100 μL/well) in triplicate and incubated overnight at 4 °C. Plates were washed three times with ELISA buffer and incubated with 100 µL peroxidase-conjugated monoclonal antihuman IgE (clone GE-1, Sigma-Aldrich, St. Louis, MO, USA) for 1 h at RT. After washing five times with ELISA buffer, 2,2′-azino-di-(3-ethylbenzthiazoline sulfonic acid) substrate solution (ABTS™-RO) (Merck KGaA, Darmstadt, Germany) was added to each well. As a result, a color reaction developed in 5 to 15 min, and the optical density (OD) values were determined at 405 nm using a SpectraMax i3x Multi-Mode Microplate Reader (Molecular Devices LLC, San Jose, CA, USA).

### 2.8. Statistics

The statistical evaluation was performed by using StatView software version 5.0 (SAS Institute, Carry, NC, USA). The non-parametric Mann–Whitney U test was used for comparing the T-cell reactivity and IgE recognition of the synthetic peptide between the allergic patients and healthy subjects. *p* values less than 0.05 were considered statistically significant. Experimental data are presented as mean ± standard error (SE).

## 3. Results

### 3.1. Pollen T-Cell and B-Cell Epitope Sequence Retrieval, Multiple Sequence Alignment, and Phylogenetic Analysis

**Figure 1:**
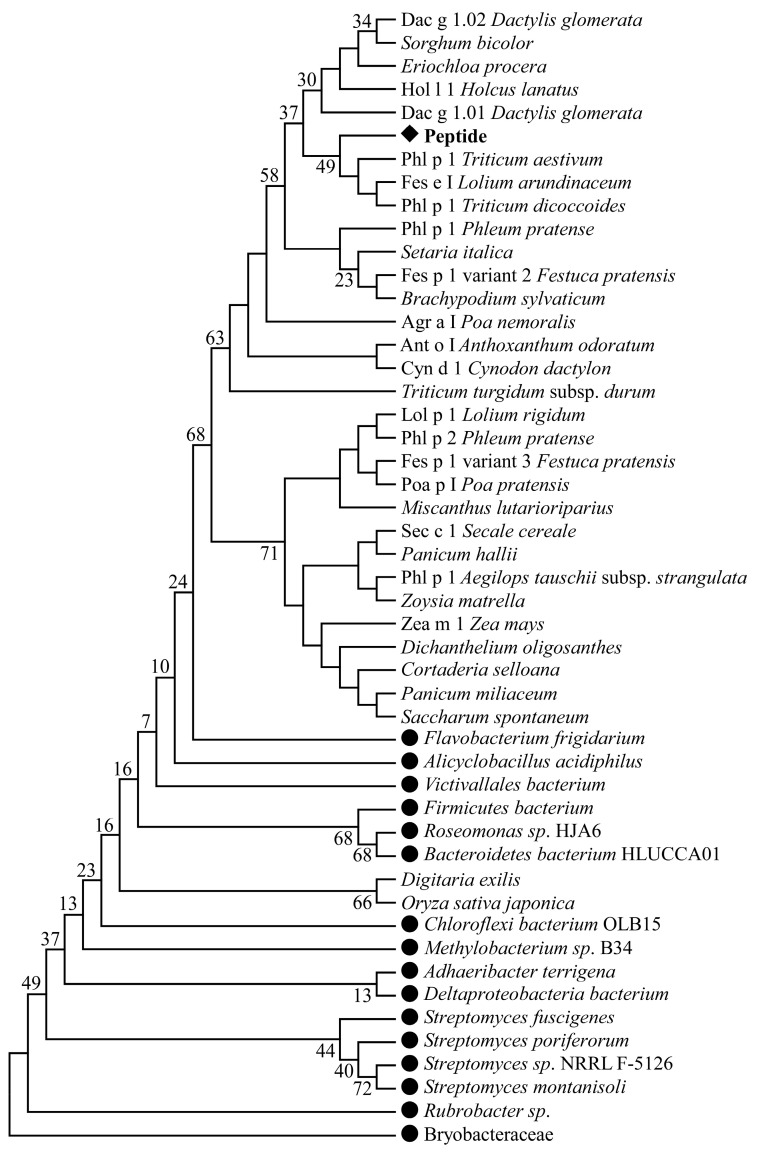
Maximum likelihood phylogenetic tree based on the GTKSEVEDVIPEGWKADTSY sequence (♦) and other amino acid sequences of allergenic and non-allergenic proteins. The numbers above branches indicate the bootstrap support from 1000 replicates. ● indicates bacterial strains.

In this study, the amino acid sequences of all T-cell and B-cell epitopes (2309 T-cell epitopes and 1010 B-cell epitopes) available in the public databases were downloaded in fasta format. Sequences alignment identified four highly conserved sequences within the investigated immune epitopes that showed similarity to distinct pollen allergens: RAEVSYVHVNGAKFI (Supplementary Materials Figure S1 and Table S1), GELQVIDKIDAAFKVAATAA (Supplementary Materials Figure S2 and Table S1), KEMGETLLRAVESYLLAHSD (Supplementary Materials Figure S3 and Table S1), and GTKSEVEDVIPEGWKADTSY (Supplementary Materials Figure S4 and Table S1). Our analyses showed that the epitope GTKSEVEDVIPEGWKADTSY was most suitable because this sequence showed similarity to the highest number of amino acid sequences of allergenic as well as non-allergenic proteins. The alignment showed that this sequence is a part of the structure of the grass group I allergens (beta-expansins): Lol-p1, Fes-p1, Phl-p1, Poa-p1, Sec-c1, Zea-m1, Ant-o1, Cyn-d1, Dac-g1, Hol-l1 (Supplementary Materials Figure S4). In addition, the epitope GTKSEVEDVIPEGWKADTSY showed binding potential to the highest number of MHC II alleles. The selected epitope has a high percentage of identity with sequences from other proteins found in bacteria such as *Roseomonas*, *Flavobacterium frigidarium*, *Deltaproteobacteria bacterium*, *Bacteroidetes bacterium*, *Bryobacteraceae bacterium*, *Muribaculaceae bacterium*, *Methylobacterium*, *Victivallales bacterium*, *Firmicutes bacterium*, *Alicyclobacillus acidiphilus*, etc. A phylogenetic tree was constructed to show the evolutionary relationship of GTKSEVEDVIPEGWKADTSY with the identified allergenic and non-allergenic proteins (Figure 1).

### 3.2. Prediction of CD8+ T-Cell Epitopes and Their MHC-I-Binding Alleles

The binding between T-cell epitopes and the specific HLA proteins (MHC I and MHC II molecules) is crucial for the cellular immune response. MHC I molecules are responsible for presentation of non-self antigens (after viral or bacterial infection) to cytotoxic T lymphocytes (CTLs) inducing cell-mediated immunity. Very often, viral and bacterial proteins can demonstrate molecular mimicry and perform cross-presentation. Seneviratne et al. found allergen-specific CD8+ T cells defining three A*0201-restricted Der p 1 (house dust mite *Dermatophagoides pteronyssinus*) CD8+ T-cell epitopes [48]. The MHC I prediction tool of the Immune Epitope Database and Analysis Resource (IEDB) was used to predict CD8+ T-cell epitopes in the GTKSEVEDVIPEGWKADTSY sequence. Based on prediction scores (the lowest percentile rank and the binding affinity of the highest numbers of alleles), only the top five epitopes were suitable for further analysis (Table 1).

The CD8+ T-cell epitope VEDVIPEGW was found to interact with ten alleles (HLA-B*44:02, HLA-B*44:03, HLA-B*40:01, HLA-C*08:02, HLA-B*57:01, HLA-A*23:01, HLA-A*24:02, HLA-C*04:01, HLA-A*01:01, and HLA-B*51:01) and it was selected for molecular docking.

### 3.3. Prediction of CD4+ T-Cell Epitopes and Their MHC-II-Binding Alleles

Likewise, five CD4+ T-cell epitopes based on their lowest percentile rank and the binding affinity of the highest numbers of alleles were predicted from the MHC II prediction tool of the IEDB server [29] for human alleles (HLA-DR, HLA-DQ, and HLA-DP) to design a peptide-based vaccine (Table 2).

The NetMHCIIpan 4.1 EL method was used by the IEDB server, which results in choosing the best immunogenic molecules. The CD4+ T-cell peptide TKSEVEDVIPEGWKA interacts with the largest number of alleles, including HLA-DQ A1*03:01/DQ B1*02:01, HLA-DQ A1*03:01/DQ B1*03:02, HLA-DQ A1*05:01/DQ B1*02:01, HLA-DQ A1*04:01/DQ B1*04:02, HLA-DQ A1*01:01/DQ B1*05:01, HLA-DR B3*01:01, HLA-DP A1*01/DP B1*04:01, and HLA-DP A1*01:03/DP B1*02:01. As shown in Table 2, the CD4+ T-cell epitope TKSEVEDVIPEGWKA and its matching HLA-class-II-binding allele DQ A1*03:01/DQ B1*02:01 were predicted as the most suitable candidates for molecular docking analysis.

### 3.4. Population Coverage Analysis of the Selected CD8+ and CD4+ T-Cell Epitopes

HLA alleles are highly polymorphic, and different HLA types are expressed at significantly different frequencies in diverse geographic regions throughout the world (in different races). The vaccines that are being designed should cover a wide range of the world population. Individual population coverage analysis for the ten selected CD8+ and CD4+ T-cell epitopes with their matching HLA alleles was calculated using the population coverage analysis tool of IEDB (http://tools.iedb.org/population/, accessed on 16 August 2023) [36]. The T-cell peptides were subjected to MHC-I- and MHC-II-based population coverage analysis in Europe and worldwide (Table 3).

It was predicted that CD8+ T-cell epitope VEDVIPEGW covers 84.57% of the population of Europe and 78.87% of the world population. CD4+ T-cell epitope TKSEVEDVIPEGWKA has a population coverage of 99.86% for Europe and 98.20% worldwide. This result indicates that the vaccine candidates against representatives of the grass group I allergens (Lol-p1, Fes-p1, Phl-p1, Poa-p1, Sec-c1, Zea-m1, Ant-o1, Cyn-d1, Dac-g1, Hol-l1) would be globally useful.

### 3.5. Identification of B-Cell Epitopes

The selected amino acid sequence (GTKSEVEDVIPEGWKADTSY) was subjected to B-cell epitope prediction (B-cell prediction tools of IEDB). The linear B-cell epitope (SEVEDVIPEGWKAD) used in this study was chosen based on various factors such as antigenicity, accessibility, hydrophilicity, flexibility, and exposed surface area. The prediction scores of Kolaskar and Tongaonkar antigenicity scale [40], Emini surface accessibility [39], Parker hydrophilicity [37], Karplus and Schulz flexibility [41], and Chou and Fashman beta turn [42] for each residue of the peptide SEVEDVIPEGWKAD are summarized in Table 4.

### 3.6. Molecular Interaction Analysis

A protein–peptide docking analysis was undertaken to assess the binding affinity between selected epitopes VEDVIPEGW (CD8+ T-cell epitope), TKSEVEDVIPEGWKA (CD4+ T-cell epitope), and SEVEDVIPEGWKAD (B-cell epitope) and their respective immune cell receptors—specific HLA proteins (HLA-B*44:03, PDB ID 4JQX and HLA-DQA1*03:01/DQB1*02:01, PDB ID 4D8P) and IgE molecules (human IgE-Fc, PDB ID 4J4P and Phl p 2-specific human IgE Fab, PDB ID 2VXQ). We downloaded all crystallographic structures of the chosen MHC alleles and IgE molecules from the RCSB PDB. The CABS-dock web server (http://biocomp.chem.uw.edu.pl/CABSdock, accessed on 26 August 2023) was used to simulate the chosen epitopes and to perform molecular docking between the epitopes and their associated receptors. In each docking case, CABS-dock predicted a set of ten best-scored models ranked according to the docking score. The best complexes were refined via their binding energy using the FireDock server [46] (Table 5).

**Figure 2:**
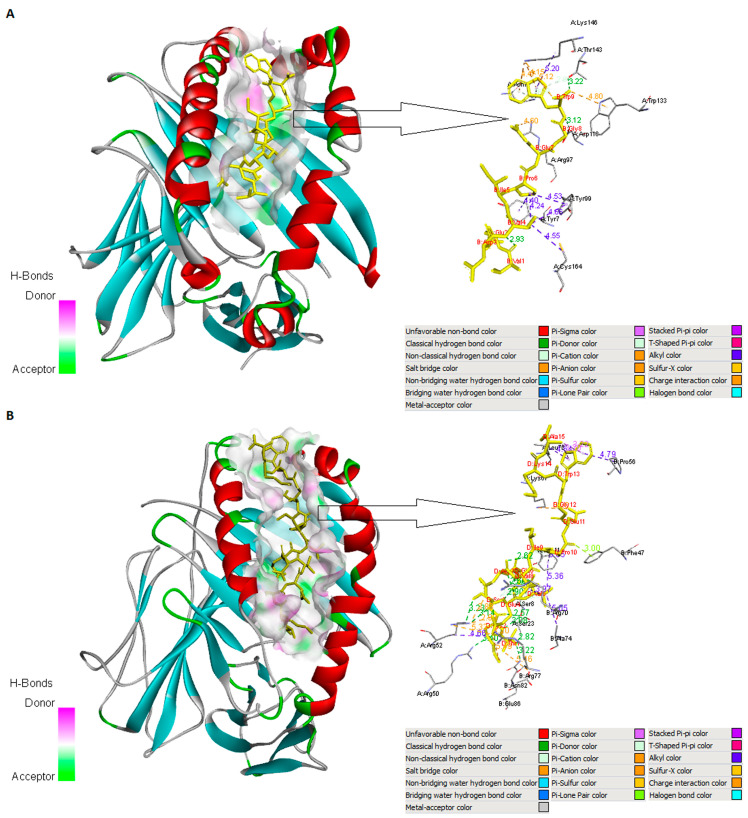
Visualization of the docking between CD8+ or CD4+ T-cell epitopes and their corresponding MHC alleles. Part (**A**) illustrates surface representation of the CD8+ T-cell epitope VEDVIPEGW (yellow color) and the MHC allele HLA-B*44:03 (blue and red color) and potential H-bonds (green sticks) and all interactions including non-polar bonds. Part (**B**) represents a schematic molecular interaction between predicted CD4+ T-cell epitope TKSEVEDVIPEGWKA (yellow color) and respective HLA allele HLA-DQA1*03:01/DQB1*02:01 (blue and red color). Different interactions between the epitopes and their corresponding MHC alleles were visualized using Discovery Studio Visualizer 2.0 and PyMOL 2.5.

**Figure 3:**
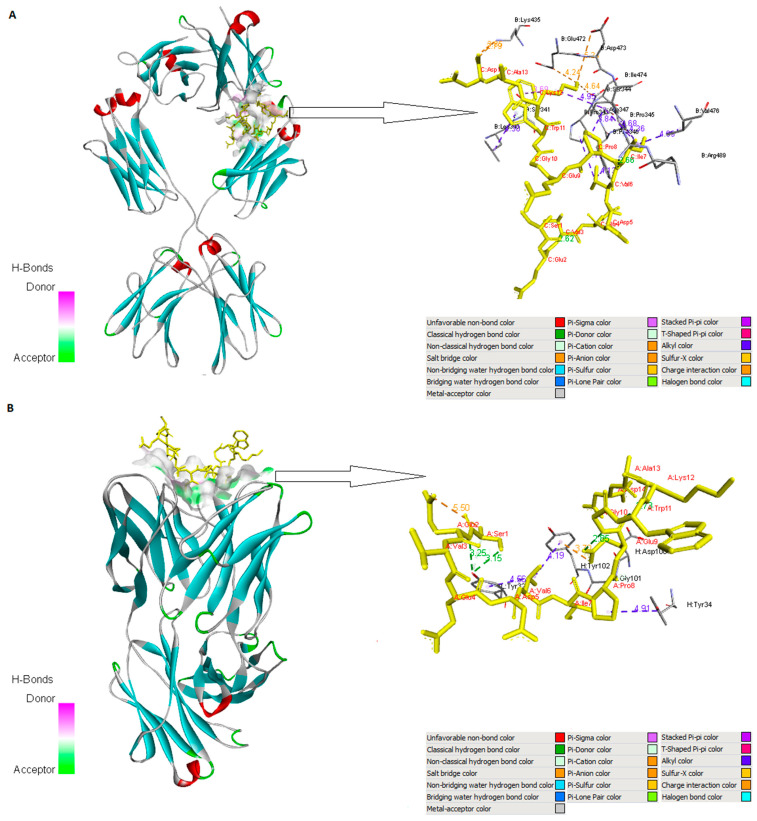
Visualization of the docking between B-cell epitope (SEVEDVIPEGWKAD) and the IgE molecules. Part (**A**) illustrates surface representation of the B-cell epitope (yellow color) and the human IgE-Fc (PDB ID 4J4P) (blue and red color) and potential H-bonds (green sticks) and all interactions including non-polar bonds. Part (**B**) represents docked complex of B-cell epitope (yellow color) with the Phl p 2-specific human IgE Fab (blue and red color). Different interactions between the B-cell epitope and the immunoglobulin E molecules were visualized using Discovery Studio Visualizer and PyMOL.

Discovery Studio Visualizer [43] and PyMOL [47] were used to visualize the interactions between the candidate ligands with their immune receptors. Highly favored molecular interactions between the CD8+ or CD4+ T-epitopes and their associated HLA alleles can be seen in Figure 2. The graphical representations of the B-cell epitope and IgE molecules are shown in Figure 3.

### 3.7. Ex Vivo Validation

**Figure 4:**
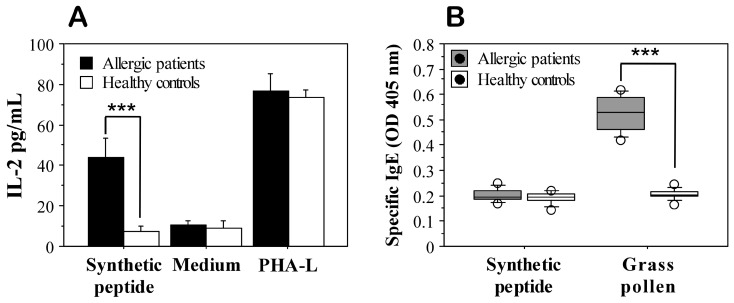
ELISA data for ex vivo T-cell response to the synthetic peptide GTKSEVEDVIPEGWKADTSY (**A**) and IgE recognition (**B**) in patients with grass pollen allergy (*n* = 8) and healthy controls (*n* = 10). IL-2 production by cultured cells in the supernatants after in vitro stimulation with the synthetic peptide for 5 days was assayed by ELISA. PHA-L was used as a positive control for T-cell stimulation. Sera were evaluated for presence of IgE antibodies that specifically recognize the synthetic peptide. As a positive control for IgE recognition, an extract from grass pollen prepared from 14 grass species was used. Results are presented as means ± SE. *** *p* = 0.0004, as determined by Mann–Whitney U test.

To validate the functional activity and safety of the studied epitope (GTKSEVEDVIPEGWKADTSY), ex vivo tests were performed with blood samples from patients with established grass pollen allergy and blood samples from healthy individuals without registered symptoms of allergic reactions. Stimulation of cells isolated from allergic patients with the synthetic peptide resulted in increased production of IL-2 (Figure 4A), demonstrating peptide presentation by MHC II molecules and T-cell activation. These results indicate that the selected epitope is functional and can activate T cells. In cells isolated from healthy individuals, no such stimulation was detected (Figure 4A).

Performed tests for the presence of specific IgE antibodies in the sera of the allergic patients that recognize the studied epitope showed a lack of reactivity (Figure 4B). This is probably due to the fact that IgE antibodies recognize a certain tertiary conformation that the relatively short peptide cannot form. This suggests the safety of the epitope-based vaccine candidate, as it is not recognized by IgE antibodies and therefore will not induce an allergic reaction.

These are only pilot tests to validate the potential and reliability of the selected peptide to be used as a basis for the development of a group 1 grass allergen vaccine. Further studies are needed to optimize and establish the specific mechanisms by which this peptide can affect specific signaling pathways to modulate the immune response.

## 4. Discussion

The immunoinformatic approach has attracted considerable attention for vaccine development against infectious and allergic diseases in recent years [49,50,51,52]. Challenges in the development of therapeutic vaccines include a selection of appropriate antigens and peptides [53]. In this study, a total of 2309 T-cell epitopes and 1010 B-cell epitopes available in public databases were aligned by the ClustalW tool of the phylogenetic software Mega X [28]. The sequences RAEVSYVHVNGAKFI, GELQVIDKIDAAFKVAATAA, KEMGETLLRAVESYLLAHSD, and GTKSEVEDVIPEGWKADTSY within the investigated immune epitopes were identified as highly conservative. The epitope KEMGETLLRAVESYLLAHSD contains the 14 mer peptide BV139 (MGETLLRAVESYLL), which has been previously described as a non-anaphylactic surface-exposed peptide of the major birch pollen allergen, Bet v 1 [54].

Akinfenwa et al. demonstrated that an early preventive administration of non-allergenic synthetic T-cell epitope-containing allergen peptide (MGETLLRAVESYLL) could be a safe strategy for the prevention of allergen-specific IgE sensitization [55]. On the other hand, the epitope GTKSEVEDVIPEGWKADTSY is a part of the structure of the grass group I allergens—Lol-p1, Fes-p1, Phl-p1, Poa-p1, Sec-c1, Zea-m1, Ant-o1, Cyn-d1, Dac-g1, Hol-l1 isolated from *Lolium rigidum*, *Festuca pratensis*, *Poa nemoralis*, *Zea mays*, *Anthoxanthum odoratum*, *Cynodon dactylon*, *Dactylis glomerata*, and *Holcus lanatus* (Figure 1). Allergens identified in one species often have homologs in other evolutionary-related species, exhibiting similar physicochemical and immunological properties. Somes allergens are restricted to Poaceae (group 1) or Pooideae (group 5) [9,56]. The epitope GTKSEVEDVIPEGWKADTSY showed its association with Phl-p2, which is a part of the grass group II allergens (Figure 1). The allergens of this group are highly homologous to group 3 and the C-terminal portion of group 1 allergens [57].

The selection of T-cell epitopes is essential for an epitope-based vaccine construction through in silico biological methods [58,59,60]. We have performed this selection according to the binding affinity of the highest numbers of alleles and the lowest percentile rank. The CD8+ T-cell peptide VEDVIPEGW was found to interact with ten alleles (HLA-B*44:02, HLA-B*44:03, HLA-B*40:01, HLA-C*08:02, HLA-B*57:01, HLA-A*23:01, HLA-A*24:02, HLA-C*04:01, HLA-A*01:01, and HLA-B*51:01), while the CD4+ T-cell peptide TKSEVEDVIPEGWKA interacts with a large number of alleles, including HLA-DQ A1*03:01/DQB1*02:01, HLA-DQ A1*03:01/DQB1*03:02, HLA-DQ A1*05:01/DQ B1*02:01, HLA-DQ A1*04:01/DQ B1*04:02, HLA-DQ A1*01:01/DQ B1*05:01, HLA-DR B3*01:01, HLA-DP A1*01/DP B1*04:01, and HLA-DP A1*01:03/DP B1*02:01, respectively. Several studies have demonstrated the importance of these receptors in the development of an effective immune response [59,60]. Although CD4+ T cells play a major role in the development of such a vaccine, Seneviratne and colleagues showed that CD8+ T cells also play a role in allergic reactions [48]. The authors demonstrated the association of these cells with the production of IFN-γ and IL-10, indicating that CD8+ T cells may provide a basis for future T-cell immunotherapy strategies through cytokine regulation [48].

Another significant factor that was considered in the selection of the epitopes for constructing the epitope-based vaccine was the population coverage. The T-cell epitopes were subjected to MHC-I- and MHC-II-based population coverage analysis in Europe and worldwide (Table 2). It was predicted that CD8+ T-cell epitope VEDVIPEGW covers 84.57% of the population of Europe and 78.87% of the world population. CD4+ T-cell epitope TKSEVEDVIPEGWKA has a population coverage of 99.86% for Europe and 98.20% worldwide. This result indicates that the selected CD8+ and CD4+ T-cell epitopes would be globally useful.

The prediction of B-cell epitopes is often less accurate than the simpler prediction of T-cell epitopes, because of their complexity [61]. Zieglmayer et al. [62] have developed a recombinant B-cell-epitope-based vaccine (BM32) for allergen-specific immunotherapy (AIT) of grass pollen allergy (i.e., Phl p 1, Phl p 2, Phl p 5, Phl p 6). The vaccine contains recombinant fusion proteins consisting of allergen-derived peptides and the hepatitis B surface protein domain preS as an immunological carrier [62]. The linear B-cell epitope SEVEDVIPEGWKAD used in this study was chosen based on various physico-chemical properties such as accessibility, hydrophilicity, flexibility, antigenicity, and exposed surface area.

In order to examine the interaction patterns of the selected B-cell epitope and CD8+ and CD4+ T-cell epitopes with their immune receptors, molecular docking was performed. Each docked epitope showed different global energy values with their corresponding receptors. The epitope TKSEVEDVIPEGWKA displayed the strongest binding affinity with its corresponding MHC allele (HLA-DQ A1*03:01/DQ B1*02:01) with a −89.59 kcal/mol value. Docking study of the CD8+ T-cell epitope (VEDVIPEGW) with HLA-B^∗^44:03 showed a very strong binding affinity to the MHC I molecule (ΔG = −72.1 kcal/mol). The human IgE-Fc (PDB ID 4J4P) showed a lower affinity with the B-cell epitope (ΔG= − 34.4 kcal/mol), while the Phl p 2-specific human IgE Fab (PDB ID 2VXQ) had the lowest binding with the B-cell epitope (ΔG = −29.9 kcal/mol) (Table 5). These interactions were achieved by van der Waals forces, pi–pi stacking, pi–alkyl, and alkyl interactions. But these are weaker interactions in comparison to the hydrogen bonds [63]. Hydrogen bonds are non-covalent forces among electronegative atoms. These bonds are formed between electronegative acceptors and donors. The preferred H-bond lengths are quite uniform, about 3.32 Å [64,65]. The formation of numerous H-bonds and non-polar interactions between the selected epitopes and their corresponding immune receptors has demonstrated that they interact properly. A total of nine hydrogen bonds were found between the CD4+ T-cell epitope (TKSEVEDVIPEGWKA) and its corresponding MHC allele (HLA-DQ A1*03:01/DQ B1*02:01): THR1-SER23 (2.82 Å), THR1-ASN82 (3.22 Å), THR1-ARG50 (3.40 Å), LYS2-SER8 (3.08 Å), LYS2-SER8 (2.57 Å), SER3-ARG52 (3.23 Å), SER3-ARG52 (3.14 Å), GLU6-SER8 (3.26 Å), and GLU6-SER8 (3.18 Å). Residues of the B-cell epitope (SEVEDVIPEGWKAD) and IgE molecule (human IgE-Fc (PDB ID 4J4P) involved in hydrogen bonding are ILE7-ARG489 (2.66 Å) and TRP11-SER341 (2.83 Å). On the other hand, the Phl p 2-specific human IgE Fab (PDB ID 2VXQ) and B-cell epitope produced a total of two hydrogen bonds, GLU2-TYR32 and SER1-TYR32, with the distance of 3.25 Å and 3.15 Å, respectively. After examination, the CD4+ T-cell epitopic peptide revealed a high affinity for the MHC class II allele and may activate an immunological response.

Our ex vivo results showed that the studied T-cell epitope is functional and can stimulate T cells. In addition, this epitope also contains a B-cell epitope, which, however, is not recognized by IgE antibodies, making it a suitable candidate for vaccine development. Despite the confirmed functionality and safety in terms of inducing an allergic reaction, at this stage our research has its limitations, since in order to establish the efficacy of the proposed epitope as a vaccine, it is necessary to conduct a number of in vitro and in vivo preclinical studies. The impact of the peptide on the production of Th1 and Th2 cytokines should be evaluated, as well as whether can it induce the production of IgG antibodies that have a therapeutic effect competing with epitope-specific IgE, whether it can it induce the generation of regulatory T cells, and whether there are any other side effects.

## 5. Conclusions

In this study, we utilized an immunoinformatics approach to design an effective epitope-based vaccine against the grass group 1 allergens (Lol-p1, Fes-p1, Phl-p1, Poa-p1, Sec-c1, Zea-m1, Ant-o1, Cyn-d1, Dac-g1, Hol-l1). After the alignment of all known pollen T-cell and B-cell epitopes from completely or partially sequenced pollen allergens available in the public databases, the peptide GTKSEVEDVIPEGWKADTSY was identified as the most promising. The proposed T- and B-cell epitopes (VEDVIPEGW, TKSEVEDVIPEGWKA, and SEVEDVIPEGWKAD) showed high sensitivity. Based on the results of performed in silico and ex vivo analyses, the proposed peptide is functional, it has the potential to induce a T-cell immune response, and it is not recognized by the IgE grass-specific antibodies. Further studies are needed to evaluate the immunotherapeutic effects of the suggested epitope-based vaccine before its clinical application.

## Figures and Tables

**Table 1:** Selected CD8+ T-cell epitopes and their corresponding MHC I alleles.

T-Cell Epitopes	Interacting MHC I Alleles	Start Position	End Position	Length of Epitope	Percentile Rank
SEVEDVIPEGWK	HLA-B*44:03 HLA-B*44:02 HLA-A*68:01 HLA-B*18:01 HLA-B*40:01 HLA-A*11:01 HLA-B*53:01	4	15	12	0.9 1.1 7.7 9.2 11.0 17.0 18.0
KSEVEDVIPEGW	HLA-B*44:03 HLA-B*58:01 HLA-B*57:01 HLA-B*44:02 HLA-B*18:01 HLA-B*53:01 HLA-B*40:01	3	14	12	0.39 0.5 0.57 0.58 1.3 4.2 5.6
SEVEDVIPEGWKA	HLA-B*44:03 HLA-B*44:02 HLA-B*18:01 HLA-B*40:01 HLA-B*40:02 HLA-B*53:01 HLA-A*68:01 HLA-A*25:01 HLA-A*68:02	4	16	13	1.2 2.0 2.5 2.9 3.1 3.3 3.7 5.4 7.5
TKSEVEDVIPEGW	HLA-B*44:03 HLA-B*58:01 HLA-B*44:02 HLA-B*57:01 HLA-B*18:01 HLA-B*53:01 HLA-B*38:01	1	14	14	0.76 0.94 4.9 6.1 8.6 9.0 11.0
**VEDVIPEGW**	HLA-B*44:02 HLA-B*44:03 HLA-B*40:01 HLA-C*08:02 HLA-B*57:01 HLA-A*23:01 HLA-A*24:02 HLA-C*04:01 HLA-A*01:01 HLA-B*51:01	6	14	9	0.02 0.03 1.0 1.3 1.5 2.26 2.8 3.1 3.2 6.7

**Table 2:** The predicted potential MHC II epitopes.

T-Cell Epitopes	Interacting MHC II Alleles	Start Position	End Position	Length	Percentile Rank
GTKSEVEDVIPEGWK	HLA-DQA1*05:01/DQB1*02:01 HLA-DQA1*03:01/DQB1*03:02 HLA-DQA1*01:01/DQB1*05:01 HLA-DQA1*04:01/DQB1*04:02 HLA-DPA1*01/DPB1*04:01 HLA-DRB1*07:03/DRB3*01:01	5	19	15	0.34 2.70 40.00 53.00 71.00 73.00
KSEVEDVIPEGWKAD	HLA-DQA1*05:01/DQB1*02:01 HLA-DQA1*03:01/DQB1*03:02 HLA-DQA1*01:01/DQB1*05:01 HLA-DQA1*04:01/DQB1*04:02 HLA-DRB1*04:21	4	18	15	0.11 2.40 23.00 26.00 39.00
**TKSEVEDVIPEGWKA**	HLA- DQA1*03:01/DQB1*02:01 HLA-DQA1*05:01/DQB1*02:01 HLA-DQA1*03:01/DQB1*03:02 HLA-DRB3*01:01 HLA-DQA1*01:01/DQB1*05:01 HLA-DQA1*04:01/DQB1*04:02 HLA-DPA1*01/DPB1*04:01 HLA-DPA1*01:03/DPB1*02:01	3	17	15	0.10 0.24 4.10 32.00 33.00 41.00 57.53 69.00
SEVEDVIPEGWKADT	HLA-DQA1*05:01/DQB1*02:01 HLA-DQA1*03:01/DQB1*03:02 HLA-DQA1*05:01/DQB1*03:01 HLA-DQA1*01:01/DQB1*05:01 HLA-DRB1*04:21	6	20	15	0.66 8.05 22.00 58.00 59.00
VEDVIPEGWKADTSY	HLA-DQA1*01:01/DQB1*05:01 HLA-DQA1*05:01/DQB1*03:01 HLA-DQA1*05:01/DQB1*02:01 HLA-DQA1*03:01/DQB1*03:02 HLA-DRB1*04:21 HLA-DRB1*04:26	2	16	15	15.00 26.00 30.00 30.00 43.00 49.00

**Table 3:** Population coverage of the selected T-cell epitopes.

Predicted T-Cell Epitopes		Population Coverage	
		World	Europe
CD8+ CD4+	SEVEDVIPEGWK GTKSEVEDVIPEGWK	44.25% 92.11%	47.68% 94.25%
CD8+ CD4+	KSEVEDVIPEGW KSEVEDVIPEGWKAD	35.60% 88.51%	42.89% 86.39%
CD8+ **CD4+**	SEVEDVIPEGWKA **TKSEVEDVIPEGWKA**	43.85% **98.20%**	49.70% **99.86%**
CD8+ CD4+	TKSEVEDVIPEGW SEVEDVIPEGWKADT	57.73% 94.13%	69.73% 92.45%
**CD8+** CD4+	**VEDVIPEGW** VEDVIPEGWKADTSY	**78.87%** 91.53%	**84.57%** 90.55%

**Table 4:** Surface accessibility (threshold = 1.000), hydrophilicity (threshold = 2.748), flexibility (threshold = 1.020), beta turn (threshold = 0.986), and antigenicity (threshold = 0.971) prediction score for each residue of B-cell epitope.

B-Cell Epitope	Emini Surface Accessibility	Parker Hydrophilicity	Karplus and Schulz Flexibility	Chou and Fashman Beta Turn	Kolaskar and Tongaonkar Antigenicity
S	1.69	5.0	1.084	0.991	0.973
E	1.956	5.614	1.064	0.977	0.972
V	1.726	4.343	1.042	0.986	1.039
E	1.38	2.386	1.021	0.834	1.071
D	1.339	2.757	1.077	0.847	1.079
V	0.791	2.757	0.998	0.847	1.079
I	1.452	3.1	1.003	0.999	1.006
P	1.285	1.557	1.016	1.03	1.012
E	1.767	1.057	1.023	0.966	1.021
G	1.105	1.771	1.028	0.989	0.975
W	1.194	3.343	1.028	1.13	0.935
K	0.995	3.786	1.035	1.05	0.912
A	1.347	3.6	1.043	1.149	0.975
D	2.007	3.629	1.053	1.089	0.976

**Table 5:** The binding properties of the docking analysis using CABS-dock web server and refined by FireDock.

PDB ID	glob	aVdW	rVdW	ACE	inside	aElec	rElec	laElec	lrElec	HB	piS	catpiS	aliph
**4JQX** (VEDVIPEGW)	−72.1	−43.5	15.1	−4.8	0.9	−13.3	17.7	−8.7	15.9	−3.8	−4.5	−1.5	−5.5
**4D8P** (TKSEVEDVIPEGWKA)	−89.6	−53.3	10.2	−0.1	1.6	−50.9	23.2	−23.5	19.5	−4.3	−5.0	0.0	−6.5
**4J4P** (SEVEDVIPEGWKAD)	−34.4	−31.4	9.7	−4.4	1.9	−69.4	14.9	−23.1	7.3	−1.9	−2.0	0.0	−5.5
**2VXQ** (SEVEDVIPEGWKAD)	−29.9	−26.7	12.0	−9.4	15.0	0.0	30.2	0.0	11.9	−2.7	−2.5	0.0	−1.5

Legend: glob—global energy, the binding energy of the complex; aVdW—softened attractive van der Waals energy; rVdW—softened repulsive van der Waals energy; ACE—atomic contact energy; inside—insideness measure; aElec, rElec—attractive and repulsive short-range Coulomb electrostatics; laElec, lrElec—attractive and repulsive long-range Coulomb electrostatics; HB—hydrogen and disulfide bonds; piS—pi–pi stacking; catpiS—cation–PI stacking; aliph—aliphatic interactions.

## Data Availability

The datasets used and analyzed during the current study are available from the corresponding author on reasonable request.

## Supplementary Materials

The following supporting information can be downloaded at: https://www.mdpi.com/article/10.3390/arm91060036/s1, Figure S1: Multiple sequence alignment of the amino acid sequence RAEVSYVHVNGAKFI showing similarity with distinct allergenic pollen proteins; Figure S2: Multiple sequence alignment of the amino acid sequence GELQVIDKIDAAFKVAATAA showing similarity with distinct allergenic pollen proteins; Figure S3: Multiple sequence alignment of the amino acid sequence KEMGETLLRAVESYLLAHSD showing similarity with distinct allergenic pollen proteins; Figure S4: Multiple sequence alignment of the amino acid sequence GTKSEVEDVIPEGWKADTSY showing similarity with distinct allergenic pollen proteins; Table S1: Percentage identity of the conserved sequences with pollen allergens.
