# Supplementary material for: In Silico Design of a New Epitope-Based Vaccine against Grass Group 1 Allergens
Source: Adv Respir Med. 2023 Nov 8;91(6):486–503. doi: 10.3390/arm91060036 (PMC10660545; doi:10.3390/arm91060036)
Supplement: Supplementary file 1 [file arm-91-00036-s001.zip › arm-2614743-Supplementary-Figs.pdf]

advances in respiratory medicine

MDPI

In Silico Design of a New Epitope-Based Vaccine against Grass Group 1 Allergens

Dzhemal Moten [1], Tsvetelina Batsalova [1], Desislava Apostolova [1], Tsvetelina Mladenova [2], Balik Dzhambazov [1] and Ivanka Teneva [2]*

☑	BAF32119.1	263	GSSNIVIEDLICGPGHGISIGSLGRENSRAEVSYVHVNGAKFIDTQNGLRIKTWQGGSGMASHIIYENVEMINSENPILI	342	[*Cryptomeria japonica*]
☑	BAC23082.1	263	GSSNIVIEDLICGPGHGISIGSLGRENSRAEVSYVHVNGAKFIDTQNGLRIKTWQGGSGMASHIIYENVEMINSENPILI	342	Cry j 2 [*Cryptomeria japonica*]
☑	BAF32142.1	233	GSSHIVIKDLICGPGHGISIGSLGRGNSRAEVSYVHVNGAKFIDTQNGLRIKTWQGGSGLASHITYENVEMVNSENPIII	312	[*Taxodium distichum*]
☑	BAF32143.1	233	GSSNITIKDLICGPGHGISIGSLGRDNSRAEVSHVHVNRAKFIDTQNGLRIKTWQGGSGLASYITYENVEMINSENPILI	312	[*Chamaecyparis obtusa*]
☑	AVW83026.1	264	GSSNITIRDLTCGPGHGMSIGSLGKGNSRSEVSFVHLDGAKFIDTQNGLRIKTWQGGSGLASHITYENVEMINAENPILI	343	Sab c 2 [*Juniperus chinensis*]
☑	ABK78768.1	211	GSSNITITDLTCGPGHGMSIGSLGKGNSRSEVSFVHLDGAKFIDTQNGLRIKTWQGGSGLASHITYENVEMVNAENPILI	290	Cup a 2 variant 1 [*Hesperocyparis arizonica*]
☑	ABK78769.1	211	GSSNITIKDLTCGPGHGMSIGSLGKGNSRSEVSFVHLDGAKFIDTQNGLRIKTWQGGSGLASHITYENVEMVNAENPILI	290	Cup a 2 variant 2 [*Hesperocyparis arizonica*]
☑	KAH9306092.1	195	GSNNIVIKDVTCGPGHGISIGSLGRDNSEAEVSNVTVDGAKFIGTQNGLRIKTWQGGSGMARDITYENIQMINAGNPIII	274	[*Taxus chinensis*]
☑	BAF32144.1	233	GSSNIQINDLTCGPGHGISIGSLGRGNSRAVVSSVHVNRAKFIDTQNGLRIKTWQGGSGIANNITYENVEMINAGNPILI	312	[*Metasequoia glyptostroboides*]

Figure S1. Multiple sequence alignment of the amino acid sequence **RAEVSYVHVNGAKFI** showing similarity with distinct allergenic pollen proteins.

☑	AAG42255.1	148	HAVKPITEEIGAAKIPAGELQIIDKIDAAFKVAATAANAAPANDKFTVFEAAFNNAIKESTGGAYDTYKSIPSLEAAVKQ	227	Hol l 5b [*Holcus lanatus*]
☑	CCI69080.1	142	HAVKPVTEEPGMAKIPAGELQIIDKIDAAFKVAATAAATAPADDKFTVFEAAFNKAIKESTGGAYDTYKCIPSLEAAVKQ	221	Ave s 5 (A) [*Avena sativa*]
☑	CAB05371.1	133	HAVKPVTEEPGMAKIPAGELQIIDKIDAAFKVAATAAATAPADDKFTVFEAAFNKAIKESTGGAYDTYKCIPSLEAAVKQ	212	Phl p 5 [*Phleum pratense*]
☑	KAK1614443.1	152	HAVKPATEEVPAAKIPTGELQIVDKIDAAFKIAATAANAAPANDKFTVFESSFNKALKECTGGAYETYKFIPSLEAAVKQ	231	[*Lolium multiflorum*]
☑	XP_047078043.1	153	HAVKPATEEVPAAKIPTGELQIVDKIDAAFKIAATAANAAPTNDKFTVFESAFNKALKECTGGAYETYKFIPSLEAAVKQ	232	Lol p 5a [*Lolium rigidum*]
☑	CCD28288.1	153	HAVKPATEEVPAAKIPTGELQIVDKIDAAFKIAATAANAAPANDKFTVFESSFNKALKECTGGAYETYKFIPSLEAAVKQ	232	Fes p 5 [*Festuca pratensis*]
☑	AAG42254.1	148	HAVKPITEETGAAKIPAGEQQIIDKIDAAFKVAATAANAAPANDKFTVFEAAFNNAIKESTGGAYDTYKSIPSLEAAVKQ	227	Poa p 5 [*Poa pratensis*]
☑	AAK62276.1	121	HAVKPAAEEVPAAKIPAGELQIVDKIDAAYKIAATAANAAPANDKFTVFEGAFNKAIKESTGGAYESYKFIPTLEAAVKQ	200	group V allergen [*Dactylis glomerata*]
☑	CAB10765.1	120	HAVKPATEEVGAAKIPAGELQIVDKIDAAFRIAATAANAAPVNDKFTVFEGAFNKAIKESTGGAYEAYKFIPSLETAVKQ	199	group V allergen [*Holcus lanatus*]
☑	CCI69081.1	159	HALKPAAEEVKA--IPAGELVVIDKIDAAFKAAATQANAAPANDKFTVFETAFNKAIKDSTGGTYETYKFVPGLEAAVKQ	236	Ave s 5 (B) [*Avena sativa*]
☑	CBG76811.1	151	HAVKPASEEVKG--VPAPQLKVVDQIDAAYRTAATAANAAPTNDKFNVFESSFNKAIKENTGGAYASYTFVPALESAVKQ	228	Sec c 5 [*Secale cereale*]

Figure S2. Multiple sequence alignment of the amino acid sequence **GELQVIDKIDAAFKVAATAA** showing similarity with distinct allergenic pollen proteins.

advances in respiratory medicine

MDPI

CAA05186.1	80	FKYNYSVIEGGPVGDTLEKISNEIKIVATPDGGCVLKISNKYHTKGNHEVKAEQVKASKEMGETLLRAVESYLLAHSDAY	159	Betv1 [*Betula pendula*]
BAB21490.1	80	FKYNYSVIEGGPVGDTLEKISNEIKIVATPDGGCVLKISNKYHTKGNHEVKAEQVKASKEMGETLLRAVESYLLSHSDAY	159	Betv1 jap2 [*Betula platyphylla var. japonica*
CAB02206.1	80	FKYNYTVIEGDVLGDKLEKVSHELKIVAAPGGGSIVKISSKFHAKGDHEVNAEKMKGAKEMAEKLLRAVESYLLAHTDEY	159	Car b 1 [*Carpinus betulus*]
ACF75001.1	77	FKYSYSVIEGGPVGDTLEKISNEIKIVATPDGGSILKISNKYHTKGDHEVKAEQIKASKEMGETLLRAVESYLL-------	150	[*Betula chichibuensis*]
ACF75005.1	77	FKYSYSVIEGGPVGDTLEKISNEIKIVETPNGGSILKISNKYHTKGDHEVKAEQIKASKEMGETLLRAVESYLL-------	150	[*Betula nigra*]
ACF74989.1	77	FKYNYSVIEGGPVGDTLEKISNEIKIVATSDGGCILKISNKYHTKGNHEVKAEQVKASKEMGETLLRAVESYLL-------	150	[*Betula costata*]
XP_059462181.1	80	FTYSYTVIEGDVLGDKLEKVCHELKIVAAPGGGSILKISSKFHAKGDHEINAEEMKGAKEMAEKLLRAVETYLLAHSAEY	159	Cor a 1 [*Corylus avellana*]
CAB02215.1	80	FKFSYTVIEGDVLGDKLEKVSLELTIVAAPGGGSILKISGKFHAKGDHEVNAEEMKGAKEMAEKLLRAVESYLLAHTAEY	159	Car b 1 [*Carpinus betulus*]
ADK39021.1	80	FKYNYTVIEGDVLGDKLEKVSHELKIVAAPGGGSILKISSKFHAKGDHEVNAEEMKGAKEMAEKLLRAVESYLLAHTAEY	159	[*Ostrya carpinifolia*]
CAB94733.1	80	FKYSYSVIEGGAVGDTLEKICNEIKIVPAPGGGSILKISNKYHTKGNHEMKAEQIKASKEKAEALFRAVESYLLAHSDAY	159	[*Betula pendula*]
WDE40133.1	81	FKYRYTVIEGGVVGDKLEKICNELRIVAAPGGGSILKISNKYHTKGGHEMNAEDIKVGKEKAEALLKAVESYLLAHSAEY	160	Cor a 1.0601 [*Corylus avellana*]
XP_059455787.1	80	FKYRYTVIEGGVVGDKLEKICNELRIVAAPGGGSILKISNKYHTKGGHEMNAEDIKVGKEKAEALLKAVESYLLAHSAEY	159	Aln g 1 [*Corylus avellana*]

Figure S3. Multiple sequence alignment of the amino acid sequence **KEMGETLLRAVESYLLAHSD** showing similarity with distinct allergenic pollen proteins.

XP_047083219.1	232	FTVRYTTEGGTKSEVEDVIPEGWKADTSYSSKk--	264	Lol p 1 [*Lolium rigidum*]
AAP96760.1	233	FTVRYTTEGGTKSEVEDVIPEGWKADTSYEAK---	264	Dac g 1.02 [*Dactylis glomerata*]
CCD28290.1	232	FTVRYTTEGGTKSEVEDVIPEGWKADTSYSAK---	263	Fes p 1 variant 2 [*Festuca pratensis*]
CCD28291.1	232	FTVRYTTEGGTKSEVEDVIPEGWKADTSYSAK---	263	Fes p 1 variant 3 [*Festuca pratensis*]
AAP96759.1	209	FTVRYTTEGGTKSEVEDVIPEGWKADTSYEAK---	240	Dac g 1.01 [*Dactylis glomerata*]
E37396	1	----YTTEGGTKAEAEDVIPEGWKADTSYE-----	26	Agr a I [*Poa nemoralis*]
P43213.1	232	FTVRYTTEGGTKTEAEDVIPEGWKADTSYESK---	263	Phl p 1 [*Phleum pratense*]
F37396	1	----YTTEGGTKAEAEDVIPEGWKVDTSYE-----	26	Poa p I [*Poa pratensis*]
CCI69079.1	213	FTVRYTTEGGTKSEVEDVIPEGWKADT--------	239	Sec c 1 [*Secale cereale*]
G37396	1	----YTTEGGKKVEAEDVIPEGWKADTSYE-----	26	Ant o I [*Anthoxanthum odoratum*]
CAA10140.1	232	FTVRYTTEGGTKVEAEDVIPEGWKADTAYESK---	263	Hol l 1 [*Holcus lanatus*]
D37396	1	----YTTEGGTKSEVEDVIPEGWK-----------	20	Fes e I [*Lolium arundinaceum*]
XP_037428368.1	236	FSVRYTTEGGTKTVVEDVIPKGWKADTSYEAKggy	270	Phl p 1[*Triticum dicoccoides*]
XP_044367192.1	236	FSVRYTTEGGTKTVVEDVIPKGWKADTSYEAKggy	270	Phl p 1[*Triticum aestivum*]
XP_040243613.2	235	FSVRYTTEGGTKTTVEDVIPKGWKADTSYEAKggy	269	Phl p 1 [*Aegilops tauschii* subsp. *strangulata*]
VAI11126.1	172	FSVRYTTEGGTKTVVEDVIPKGWKADTSYEAKggy	206	[*Triticum turgidum* subsp. *durum*]
XP_002466019.1	235	VTVQITTEGGTKTAYEDVIPEGWKADTTYTAK---	266	[*Sorghum bicolor*]
XP_004986062.1	234	IAIRLTSEGGTKLEQDDVIPEGWKPDTVYTSKlqf	268	[*Setaria italica*]
KAF8673784.1	234	IAVRLTSEGGTTIEQEDAIPEGWKADTIYTSKiqf	268	[*Digitaria exilis*]
RLM78148.1	234	ISIRVTSEGGKKLEQEDVIPEGWKPDTLYPSKlqf	268	[*Panicum miliaceum*]
PVH33379.1	115	ISIRITSEGGKTLEQEDVIPEGWKPDTLYPSKlqf	149	[*Panicum hallii*]
ABD79095.1	221	LTVRLTTEGGTKSVYDDVIPANWKANTAYTAK---	252	Zea m 1 [*Zea mays*]
AAL14079.1	228	FTIRLTSESGGHVEQEDVIPEDWKPDTVYKSKiqf	262	Cyn d 1 [*Cynodon dactylon*]

Figure S4. Multiple sequence alignment of the amino acid sequence **GTKSEVEDVIPEGWKADTSY** showing similarity with distinct allergenic pollen proteins.
