# Supplementary material for: In Silico Design of a New Epitope-Based Vaccine against Grass Group 1 Allergens
Source: Adv Respir Med. 2023 Nov 8;91(6):486–503. doi: 10.3390/arm91060036 (PMC10660545; doi:10.3390/arm91060036)
Supplement: Supplementary file 1 [file arm-91-00036-s001.zip › arm-2614743-Supplementary-Table-S1.pdf]

advances in respiratory medicine

MDPI

Article

In Silico Design of a New Epitope-Based Vaccine against Grass Group 1 Allergens

Dzhemal Moten [1], Tsvetelina Batsalova [1], Desislava Apostolova [1], Tsvetelina Mladenova [2], Balik Dzhambazov [1] and Ivanka Teneva [2]*

Table S1. Percentage identity of the conserved sequences with pollen allergens.

Peptide sequence: RAEVSYVHVNGAKFI		
**Accession**	**Description**	**Percentage identity**
BAF32119.1	[*Cryptomeria japonica*]	100.00%
BAC23082.1	Cry j 2 [*Cryptomeria japonica*]	100.00%
BAF32142.1	[*Taxodium distichum*]	100.00%
BAF32143.1	[*Chamaecyparis obtusa*]	86. 67%
AVW83026.1	Sab c 2 [*Juniperus chinensis*]	73.33%
ABK78768.1	Cup a 2 var. 1 [*Hesperocyparis arizonica*]	73.33%
ABK78769.1	Cup a 2 var. 2 [*Hesperocyparis arizonica*]	73.33%
KAH9306092.1	[*Taxus chinensis*]	78.57%
BAF32144.1	[*Metasequoia glyptostroboides*]	80.00%
**Peptide sequence: GELQVIDKIDAAFKVAATAA**		
**Accession**	**Description**	**Percentage identity**
AAG42255.1	Hol l 5b [*Holcus lanatus*]	95.00%
CCI69080.1	Ave s 5 (isoallergen A) [*Avena sativa*]	95.00%
CAB05371.1	Phl p 5 [*Phleum pratense*]	95.00%
KAK1614443.1	[*Lolium multiflorum*]	85.00%
XP_047078043.1	Lol p 5a [*Lolium rigidum*]	85.00%
CCD28288.1	Fes p 5 [*Festuca pratensis*]	85.00%
AAG42254.1	Poa p 5 [*Poa pratensis*]	85.00%
AAK62276.1	group 5 isoallergen 1 [*Dactylis glomerata*]	80.00%
CAB10765.1	group V allergen [*Holcus lanatus*]	80.00%
CCI69081.1	Ave s 5 (isoallergen B) [*Avena sativa*]	85.00%
CBG76811.1	Sec c 5 [*Secale cereale*]	73.16%
**Peptide sequence: KEMGETLLRAVESYLLAHSD**		
**Accession**	**Description**	**Percentage identity**
CAA05186.1	Betv1 [*Betula pendula*]	100.00%
BAB21490.1	Bet vI jap2 [*Betula platyphylla var. japonica]*	95.00%
CAB02206.1	Car b 1 [*Carpinus betulus*]	85.00%
ACF75001.1	[*Betula chichibuensis*]	100.00%
ACF75005.1	[*Betula nigra*]	100.00%
ACF74989.1	[*Betula costata*]	100.00%
XP_059462181.1	Cor a 1 isoforms 5, 6, 11 and 16 [*Corylus avellana*]	84.21%
CAB02215.1	Car b 1 [*Carpinus betulus*]	88.89%

advances in respiratory medicine

MDPI

ADK39021.1	[*Ostrya carpinifolia*]	88.89%
CAB94733.1	[*Betula pendula*]	80.00%
WDE40133.1	[*Corylus avellana*]	78.95%
XP_059455787.1	Aln g 1-like [*Corylus avellana*]	78.95%
**Peptide sequence: GTKSEVEDVIPEGWKADTSY**		
**Accession**	**Description**	**Percentage identity**
XP_047083219.1	Lol p 1-like [*Lolium rigidum*]	100.00%
AAP96760.1	Dac g 1.02 [*Dactylis glomerata*]	100.00%
CCD28290.1	Fes p 1 var 2 [*Festuca pratensis*]	100.00%
CCD28291.1	Fes p 1 var 3 [*Festuca pratensis*]	100.00%
AAP96759.1	Dac g 1.01 [*Dactylis glomerata*]	100.00%
E37396	Agr a I [*Poa nemoralis*]	90.00%
P43213.1	Phl p 1 [*Phleum pratense*]	90.00%
F37396	Poa p I [*Poa pratensis*]	85.00%
CCI69079.1	Sec c 1 [*Secale cereale*]	100.00%
G37396	Ant o I [*Anthoxanthum odoratum*]	85.00%
CAA10140.1	Hol l 1 [*Holcus lanatus*]	85.00%
D37396	Fes e I type B [*Lolium arundinaceum*]	100.00%
XP_037428368.1	Phl p 1 [*Triticum dicoccoides*]	85.00%
XP_044367192.1	Phl p 1 [*Triticum aestivum*]	85.00%
XP_040243613.2	Phl p 1 [*Aegilops tauschii subsp. strangulata*]	85.00%
VAI11126.1	[*Triticum turgidum subsp. durum*]	85.00%
XP_002466019.1	[*Sorghum bicolor*]	80.00%
XP_004986062.1	[*Setaria italica*]	80.00%
KAF8673784.1	[*Digitaria exilis*]	80.00%
RLM78148.1	[*Panicum miliaceum*]	80.00%
PVH33379.1	[*Panicum hallii*]	81.25%
ABD79095.1	Zea m 1 [*Zea mays*]	75.00%
AAL14079.1	Cyn d 1 [*Cynodon dactylon*]	75.00%
